# Multiple dimension equity assessment of walking for visual and physical access to central parks across 27 Yangtze River Delta cities

**DOI:** 10.1038/s41598-026-47920-w

**Published:** 2026-04-13

**Authors:** Ziqun Lin, Lifei Wang, Xu Zhen

**Affiliations:** College of Landscape Architecture, Nanjing Forestry University, Nanjing, China

**Keywords:** Walkability, Visual perceptibility, Street view imagery (SVI), Landscape analytics, Yangtze River Delta, Urban agglomeration, Environmental social sciences, Environmental studies, Geography, Geography

## Abstract

The Yangtze River Delta (YRD), as a globally significant megacity region, faces intense land constraints and growing demands for equitable access to high-quality urban parks. However, traditional planning often prioritizes physical proximity while overlooking the sensory experience, leading to a disconnect between where residents can “reach” and what they can “see.” To bridge this gap, this study develops an integrated framework that couples pedestrian potential with visual perception. By integrating GIS-based pedestrian network analysis and FCN-driven semantic segmentation of street-view image, we evaluated the central parks of 27 cities across the YRD. The results demonstrate that: (1) Urban scale is closely linked to service equity, with 37% of cities exhibiting a distinct accessibility disadvantage relative to the regional average. (2) Large cities tend to dominate in green perception (GVI), whereas small and medium-sized cities are characterized by stronger blue perception (BVI). (3) A significant spatial divergence exists between physical walkability and blue-green perception, manifesting into four distinct spatial typologies at the urban scale. (4) Areas theoretically expected to have high accessibility and perception—such as waterfronts or park edges—underperform in reality. These findings suggest that theoretical attractiveness does not naturally translate into functional accessibility without intentional street-level integration. Achieving park equity requires a dual focus on both sensory experience and physical access. Consequently, this study proposes a targeted urban renewal framework to assist managers in implementing type-specific micro-interventions, providing a scalable model for achieving sensory-physical synergy in land-constrained megacity regions.

## Introduction

Urban regions have become a primary form of regional development and governance. As China’s most economically advanced, densely populated, highly industrialized, and dynamic area, the Yangtze River Delta (YRD) is evolving into a globally significant megacity region. While characterized by numerous lakes and the highest hydrographic density in China, the region simultaneously faces immense pressure from rapid urbanization and inequitable resource allocation^1,2^. Against the global backdrop of increasing emphasis on regional coordinated development and sustainable urban governance, measuring urban green spaces and their utilization efficiency has become profoundly valuable^3^. Urban parks—particularly medium-to-large parks in central districts—serve as the core components of urban green infrastructure and act as a vital medium for daily human-nature interaction^4^. Functioning not only as “urban green living rooms” but also as physical anchors for urban identity, public life, and cultural formation^5,6^, the effectiveness of these spaces depends heavily on residents’ opportunities to access the parks and their perception of the surrounding environment^7^.

Despite the widely recognized multifaceted values of urban parks, their equitable accessibility has not been synchronized with the pace of rapid urban expansion. As essential public resources, the spatial distribution and service efficiency of urban parks remain uneven across different urban areas, social groups, and spatial scales; such inequality is increasingly becoming a critical bottleneck for sustainable urban development^4,8^. Existing research indicates that high-income groups in city centers typically enjoy better access to high-quality park resources, whereas low-income groups are often concentrated in peripheries or rural–urban fringes with underdeveloped infrastructure, thereby exacerbating spatial resource maldistribution and socio-economic segregation^8^. Consequently, while urban fringes are frequently identified as vulnerable areas for park accessibility and resource allocation, city centers are generally perceived to possess resource aggregation and service advantages.

However, with the massive population influx accompanying rapid urbanization, land available for park construction has become increasingly scarce^9,10^. Even within high-income central communities, residents’ opportunities to access parks are constrained to varying degrees by high-density development and limited land resources^11^. Inequality within city centers appears to intensify during rapid urbanization, yet it is often obscured by overarching economic trends^12^. Such service inequities also persist across different urban scales and regions. Constrained by institutional frameworks and financial resources, small cities often lag behind large cities in park planning and allocation, leading to potentially more pronounced localized inequalities^9^. Globally, megacities at different developmental stages face unique structural issues: ethnic stratification creates access disparities in developed nations, while megacities in developing countries often suffer from severe resource imbalances following periods of rapid expansion^4,13^. Although prior studies have addressed the disproportionate distribution of park services within and between cities, a systematic exploration of these disparities at the urban agglomeration scale remains insufficient, with a noticeable lack of comparative research.

Under these circumstances, investigating the inequalities in services provided by urban parks across different urban spaces and scales within a megacity region is of paramount importance. Current research evaluating the service benefits of urban parks primarily focuses on two core dimensions: physical accessibility and visual perception quality. Physical accessibility represents the foundational level, measuring the proximity of parks to residential areas and the ease with which residents can walk to and enter a given park. In contrast, visual perception quality reflects the sensory information—such as the visibility of blue-green features—provided by the park and its surroundings, which directly shapes the subjective experience of environmental quality. Extensive research suggests that higher accessibility leads to increased park usage. However, Zhang and Chen^14^ demonstrated that environmental perception possesses stronger explanatory and predictive power for park use than physical reach alone. When navigating to a park, residents consider more than just distance; they prioritize the subjective sensory experience offered by the surrounding environment. Particularly in the post-pandemic era, this intensified psychological reliance on nature has made residents more sensitive to the visual perception of blue-green environments. In the YRD region, where dense hydrographic networks interweave with green spaces, these blue-green infrastructures serve as the primary carriers of visual perception, significantly influencing the service efficacy of urban parks. Essentially, the benefits of a park depend not only on its “existence” (physical reach) but also on whether its environmental quality can be effectively “perceived” by its users.

Nevertheless, does “reaching” a park necessarily equate to a high-quality “seeing” experience? Physical reach and visual perception often lack synchronicity, a phenomenon that may vary significantly depending on urban environmental characteristics, city scales, and regional contexts. Currently, integrated assessments combining both pedestrian accessibility (Physical Reach) and visual perception (Visual See) remain limited. A single-dimensional evaluation fails to fully capture the complex inequalities inherent in urban park services.

In this study, we adopt a comprehensive evaluation framework that integrates physical and perceptual dimensions, specifically focusing on the pedestrian accessibility and visual perception characteristics of parks and their surrounding blue-green spaces. To bridge the research gap at the regional scale, this study shifts the focus from individual cities to the YRD urban agglomeration, selecting central parks across 27 prefecture-level cities as subjects to explore service equity disparities across different urban scales and spatial contexts. We utilize GIS-based pedestrian network analysis to quantify physical accessibility. Simultaneously, we employ a street-view imagery recognition method based on Fully Convolutional Networks (FCN) to extract the visual features of blue-green spaces, thereby measuring the visual perception quality^15^. By integrating these two dimensions, this research investigates the dual relationship—including spatial correlations and potential asynchrony—between the walking environment’s physical reachability and its visual sensory appeal. Specifically, we defines the streetscapes within the park’s 15-min walking catchment as the primary analytical boundary, rather than the internal park space itself. This design is grounded in the theory that urban green services are not confined to the park’s internal space but extend to the perceptual journey of the user. Within this framework, the visual perception of the streets surrounding a park acts as a critical determinant of service equity, as it shapes the resident’s psychological proximity and willingness to utilize the resource^16^. Within this context, we address the following research questions:

i. *Disparities in walkability* What are the significant variations in pedestrian accessibility (“Walking to Reach”) to central parks across cities of different scales within the YRD urban agglomeration?
ii. *Diversities in perception* How do residents’ visual perceptions of blue-green environments (“Walking to See”) within and around central parks differ across various urban contexts?
iii. *Mismatch between reach and perception* Is there a spatial asynchrony between physical access to parks and the visual perception of their environmental quality? Furthermore, what are the distinct characteristics of this mismatch—where parks are “reachable” but lack “visual appeal”—across different urban scales?

## Literature review

### Urban central parks in the Yangtze River Delt

As one of the world’s six major megalopolises, the Yangtze River Delta (YRD) represents China’s most dynamic and innovative urban agglomeration. Centered around the Yangtze River, this region has evolved a spatial structure deeply intertwined with its extensive hydrological networks. These river systems serve as critical natural assets and cultural mediators, fundamentally shaping the spatial cognition of regional residents^17^. Leveraging this ecological foundation, the YRD has demonstrated significant potential in fostering blue-green integration and developing park-city models. Within this context, a water-centric park system has emerged, characterized by a landscape pattern where parkland and water bodies interpenetrate. These central parks are more than mere recreational spaces; they are representative anchors of urban identity, often serving as the primary medium through which residents experience and perceive the city^18^.

In dense urban cores, central parks occupy a prioritized position as vital nodes of urban form and essential resources for public well-being and spatial equity^5,6,19^. However, these high-density environments impose significant structural constraints on park performance. Many legacy green spaces in central areas suffer from insufficient scale, fragmented structures, and mono-functional designs^16^. Furthermore, historical planning often prioritized compensatory and functional goals over systemic integration, making it difficult to meet the contemporary demands of high-quality urban development^20^. Intense land-use competition has further compressed these spaces, preventing their physical accessibility and service benefits from being fully realized^1,2^.

From a regional perspective, urban central parks function as dual anchors within both the ecological and social networks of the urban agglomeration. Despite their importance, existing research has predominantly focused on individual cities to analyze park distribution and functionality^9,21,22^. While these studies provide valuable insights into internal resource allocation, they lack a systemic, cross-city assessment of park. Consequently, regional disparities and imbalances remain insufficiently explored. Moreover, current scholarship leans heavily toward the physical dimension of accessibility—the “access”—while empirical research on the perceptual dimension remains limited. There is, therefore, a pressing need to investigate the morphological characteristics and service equity of central parks within regional hydrological frameworks across multiple scales. By integrating physical reach with visual perception, this study aims to provide a theoretical foundation for optimizing green infrastructure and urban spatial governance at the metropolitan scale.

### Access to parks and visual perception

The potential for residents to utilize urban parks is governed by two decisive factors: the physical ease of entry and the perceptual quality of the journey. To function effectively as ecological and social infrastructures, parks must not only be spatially convenient but also perceptually engaging during the approach^16^. Traditionally, physical accessibility was measured through static metrics such as Euclidean distance and buffer analysis. However, as spatial data and network modeling have advanced, the focus has shifted toward dynamic path analysis within street networks. This evolution emphasizes functional “reachability” over mere “spatial proximity,” utilizing indicators such as the shortest street-network paths and Pedestrian Route Directness (PDR) to capture the rationality of pedestrian permeability^23^. While physical accessibility quantifies the separation between users and destinations, visual perception—defined as an individual’s subjective experience of the walking environment—has proven more significant in predicting actual usage^7^. Despite its importance, perception is rarely evaluated with the same rigor as physical metrics, a gap that is particularly acute in the water-centric spatial structure of the Yangtze River Delta^7^. Walkability is constrained by micro-structures such as water fragmentation and bridge connectivity, while blue spaces adjacent to pedestrian paths significantly enhance visual intimacy and psychological proximity^24,25^.

Research indicates that the distribution of perceptual quality and physical walkability varies significantly across urban scales^8,26^. In megacities like Shanghai, urban renewal has improved walkability in peripheral areas, yet central park cores often experience a “walkability dip” due to high-density infrastructure^18,20^. In contrast, residents in small-to-medium cities face limited opportunities for physical access despite often residing in more naturalistic environments^24^. Perceptually, high-density development and building shadows in megacity centers can obstruct street-level visibility, leading to a decline in visual quality^22^. Conversely, while SMCs offer higher landscape continuity, their perceptual potential is often hindered by fragmented greenery and a lack of clear directional cues, which limits the “perceived reachability” of blue-green assets^17,27^. This duality suggests that park equity cannot be understood through a single lens; it requires a systematic comparison across different urban hierarchies. While conventional assessments have traditionally relied on remote sensing and GIS data, these approaches often fail to capture street-level details. Recently, Street View Imagery (SVI) and recognition technologies have emerged as transformative tools. By simulating the pedestrian’s perspective, these methods quantify the visual quality of the transit environment (GVI/BVI), providing a robust tool to bridge the gap between traditional spatial data and the lived experience of the urban streetscape^28,29^.

## Materials and methods

### Study area

**Fig. 1:**
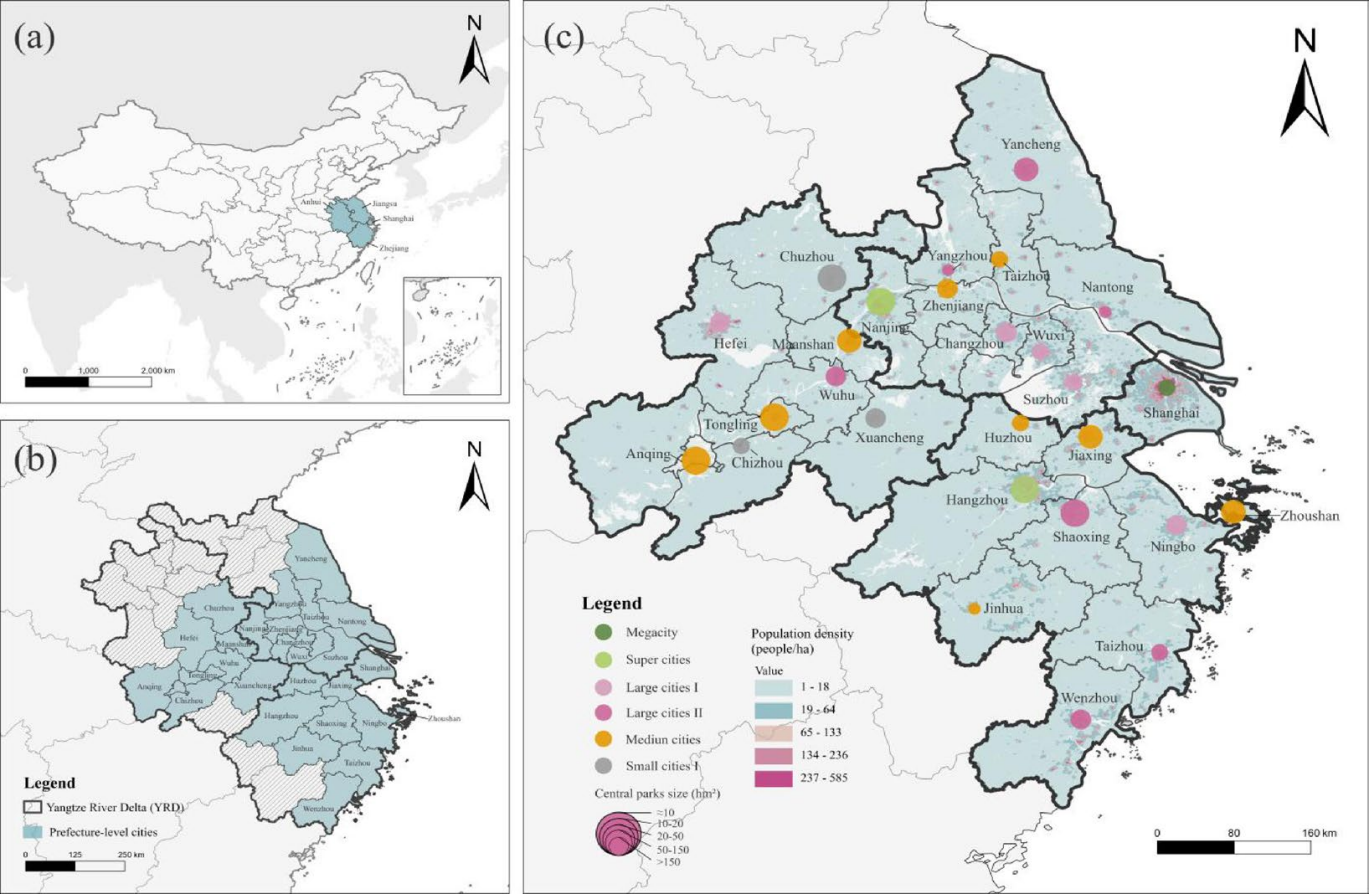
Geographic distribution and basic characteristics of 27 urban central parks. (**a**). Geographic location of the Yangtze River Delta; (**b**). 27 provincial-level cities in the YRD; (**c**). Geographic distribution of 27 urban central parks and their corresponding city scales. (Map created by the authors using ArcGIS Pro 3.6 (Esri Inc.). The administrative boundaries and base map information for China and the Yangtze River Delta were derived from the Esri World Topographic Map and open-source geographic data.)

The Yangtze River Delta (YRD) urban agglomeration stands as one of China’s most prominent economic engines and is rapidly evolving into a world-class metropolitan region. Centered on Shanghai, the YRD encompasses the provinces of Zhejiang, Jiangsu, and Anhui (Fig. 1a). This study focuses on the central area defined by the *Yangtze River Delta Regional Integration Development Plan (2019)*, covering 27 prefecture-level cities (Fig. 1b). Cities within the YRD exhibit a pronounced developmental gradient. According to the official classification based on permanent resident populations, these 27 cities are categorized into six tiers: megacities (> 10 million), super-large cities (5–10 million), large cities I (3–5 million), large cities II (1–3 million), medium cities (0.5–1 million), and small cities I (0.2–0.5 million). For each city, one centrally located park with free admission was selected as a representative case study, totaling 27 central parks (Fig. 1c). To ensure sample representativeness and mitigate potential selection bias, we employed a systematic selection protocol based on functional intensity rather than random sampling. The selection criteria for the parks were as follows:

i. *Locational centrality* Parks must be situated within the urban core. In polycentric cities, the “primary center” was identified as the area of peak population intensity according to Baidu Heat Maps (Fig. 1c). In cities without ring roads, parks were identified near areas of peak intensity as indicated by Baidu Heat Maps (Fig. 1c), ensuring alignment with the functional city center.
ii. *Public accessibility* Only parks with free admission were included, as entrance fees hinder the principles of public equity and spatial openness.
iii. *Multifunctionality* Selected parks must function as comprehensive green spaces, integrating natural landscape elements with diverse facilities for outdoor recreation, leisure, and public services.
iv. *Spatial scale* Adhering to the *Standard for Classification of Urban Green Space (CJJ/T 85-2017)*, a minimum area threshold of 10 hm^2^ was established to ensure that the selected cases represent “Comprehensive Parks” capable of providing significant ecological and social services. Smaller parks (e.g., pocket parks or community gardens) were excluded to maintain methodological consistency across the 27 cities, as their service radii and functional capacities differ fundamentally from those of central comprehensive parks.

### Datasets

Our analytical framework integrates multiple spatially explicit datasets, encompassing the spatial boundaries of central parks, serviced residential buildings, calculated shortest pedestrian routes, Pedestrian Directness Ratios (PDR), and Street View Imagery (SVI).

#### Central parks

The park dataset primarily encompasses: (1) access points (entrances), (2) spatial boundaries, (3) land and water areas, and (4) other categorical attributes. Spatial data for the 27 selected parks were retrieved from Baidu Maps via automated Python-based web scraping.

In the Chinese urban context, central parks are typically enclosed by fences or walls, with designated gates for management purposes. However, conventional datasets or path-based crawling algorithms frequently fail to account for numerous informal or inconspicuous access points^30^. Such omissions can introduce significant bias into the assessment of park accessibility and service coverage. To mitigate this, our study utilized Street View Imagery (SVI) to visually identify and verify potential informal entrances, thereby refining the accuracy of our accessibility models.

#### Residential buildings serviced

Service areas or catchments defined by Euclidean (linear) distance—though frequently adopted in planning proposals for their simplicity—tend to overestimate the actual resident population served, as pedestrian accessibility is fundamentally governed by real-world walking routes. To ensure a more realistic assessment, we utilized residential building POI data, identifying all residential points within a 1500-m network distance (walking distance) from the nearest park entrance. This threshold assumes that residents within these catchments can conveniently access the parks on foot. Furthermore, the discrepancy between service areas measured via Euclidean versus network distances serves as a critical indicator of pedestrian permeability and urban fabric connectivity.

#### Shortest routes to the parks

In this study, routes generated via the Baidu Map API constitute the primary analytical basis for calculating accessibility metrics, whereas the digitized pedestrian network was employed for park baseline statistics and the calibration of API origins and destinations, ensuring that all simulated trips terminated at verified, functional park access points. As the core routing engine, the Baidu Map API effectively integrates complex urban ‘routing semantics’—such as gated communities, 3D pedestrian structures, and real-time road constraints—while providing the temporal data fidelity necessary for a more accurate analysis of urban walking accessibility. Prior to measuring the shortest walking distances (ND) from residential POIs, a 1,500-m Euclidean buffer (ED) was constructed to pre-filter the data, thereby streamlining the computational process. This Euclidean pre-filtering serves as a conservative and reliable sampling threshold; since the Euclidean distance represents the theoretical minimum path between any two points (ED ≤ ND), any residential building with a walking network distance of 1500 m or less must inherently fall within this 1500-m Euclidean radius. This approach ensures that no potentially serviced buildings are omitted while maintaining computational efficiency.

We have adopted a multi-level walking service distance of 5, 10, and 15 min (corresponding to 500 m, 1000 m, and 1500 m) in our study. This selection is based on the life circle classification established in the “Standard for Planning and Design of Urban Residential Areas” (GB 50,180–2018). Additionally, the 1500 m threshold accounts for the significant attractiveness and service capacity of large-scale parks, aligning with the urban park accessibility standards proposed by De Chiara and Koppelman^31,32^.

#### Pedestrian directness ratio (PDR)

For each identified route, we calculated both the Euclidean (linear) distance and the network distance (the latter formally termed “route distance” in GIS spatial analysis). The Pedestrian Directness Ratio (PDR) was then derived by dividing the network distance by the corresponding Euclidean distance. By definition, the PDR is always greater than or equal to 1, as real-world urban configurations rarely facilitate perfectly straight pedestrian movement. A higher PDR value signifies a more circuitous walking path, serving as a proxy for physical barriers, limited park permeability, and the overall complexity of the surrounding built environment. The Pedestrian Directness Ratio (PDR) is defined as:

$$PDR_{{{\mathrm{ij}}}} = \frac{{S{}_{ij}}}{{C_{ij} }}$$

- *PDR*_*ij*_ is the pedestrian directness ratio between origin* i* and destination *j*;
- *i* denotes the origin, representing the location of a specific residential POI;
- *j* represents the destination, referring to the location of a park entrance;
- *S*_*ij*_ denotes the distance of the shortest walking path (network distance) between *i* and *j*;
- *C*_*ij*_ represents the Euclidean distance (crow-fly distance) from *i* to *j.*

#### Number of turns (NOT) and number of road crossings (NRC)

To provide a more comprehensive assessment of pedestrian permeability, we introduced the Number of Turns (NOT) and the Number of Road Crossings (NRC) as independent indicators to complement the spatial efficiency measured by PDR.The Number of Turns (NOT) is utilized to quantify the cognitive complexity of a walking route, calculated by counting the nodes along each shortest path where the directional change exceeds 45°. A higher NOT value indicates a more tortuous route that necessitates greater cognitive mapping effort for pedestrians.

The Number of Road Crossings (NRC) serves as a proxy for pedestrian safety and traffic interference; it is determined by identifying the intersection points between the digitized pedestrian paths and the motorized road network, with each crossing of a vehicular lane recorded as one NRC. These two metrics are treated as independent descriptive indicators rather than being aggregated into a weighted composite index with PDR. By evaluating NOT and NRC alongside PDR, this multi-dimensional approach allows for the identification of routes that may be geometrically direct yet cognitively demanding or safety-compromised, thereby offering a more nuanced interpretation of urban connectivity and walking comfort.

#### Green view index (GVI)

The Green View Index (GVI) quantifies the proportion of visible green vegetation at street level and is a widely recognized metric for assessing visual greenness exposure^33^. The GVI for each sampling site is derived by calculating the average proportion of green pixels across four street-view images captured in different cardinal directions^34^. The selection of GVI thresholds in this study is grounded in the classification standards proposed by Orihara et al., which categorize visual greenery perception into five distinct levels. Specifically, a GVI of 25% is identified as the critical benchmark for “good” visual quality. Accordingly, we adopted 25% as the target line for our analysis: sites with GVI ≤ 25% are classified as having poor or insufficient visual perception, while those with GVI > 25% are considered to provide superior or high-quality green exposure. The calculation of GVI is defined as follows:

$$GVI_{{\mathrm{j}}} = \frac{{\sum\limits_{i = 1}^{4} {Area_{g\_i} } }}{{\sum\limits_{i - 1}^{4} {Area_{T\_i} } }} \times 100\%$$

- *GVI*_*j*_ denotes the green view index at a specific sampling site *j*;
- *i* denotes the *i*^*th*^ street-view image captured at the site, corresponding to one of the four cardinal directions (0°, 90°, 180°, 270°);
- *j* represents the unique spatial index of each sampling site along the pedestrian network;
- *Area*_*g_i*_ is the total number of pixels identified as green vegetation in the *i*^*th*^ street-view image;
- *Area*_*T_i*_ denotes the total number of pixels in the *i*^th^ street-view image.

#### Blue view index (BVI)

Drawing on established methodologies, this study introduces the Blue View Index (BVI) to evaluate visual exposure to blue spaces, specifically focusing on visible water bodies^35^. Similar to the GVI, the BVI measures the visual prominence of blue features within the urban streetscape. However, given that the proportion of blue space captured in street-view imagery is inherently significantly lower than that of green vegetation, we adopted a binary classification for blue perception. Sites are categorized into two levels: “perceivable blue space” (BVI > 0) and “poor blue perception” (BVI = 0). This threshold ensures that even marginal hydrological visibility is accounted for, reflecting the unique water-town characteristics of the YRD region while acknowledging the structural constraints of blue space exposure in dense urban streetscapes.

$$BVI_{{\mathrm{j}}} = \frac{{\sum\limits_{i = 1}^{4} {Area_{{{\mathrm{b}}\_i}} } }}{{\sum\limits_{i - 1}^{4} {Area_{T\_i} } }} \times 100\%$$

- *BVI*_*j*_ denotes the blue view index at a specific sampling site *j*;
- *i* denotes the *i*^*th*^ street-view image captured at the site, corresponding to one of the four cardinal directions (0°, 90°, 180°, 270°);
- *j* represents the unique spatial index of each sampling site along the pedestrian network;
- *Area*_*b_i*_ represents the total number of blue pixels (identified as water bodies) in the image captured from direction *i*;
- *Area*_*T_i*_ denotes the total number of pixels in the *i*^th^ street-view image.

### Methods

**Fig. 2:**
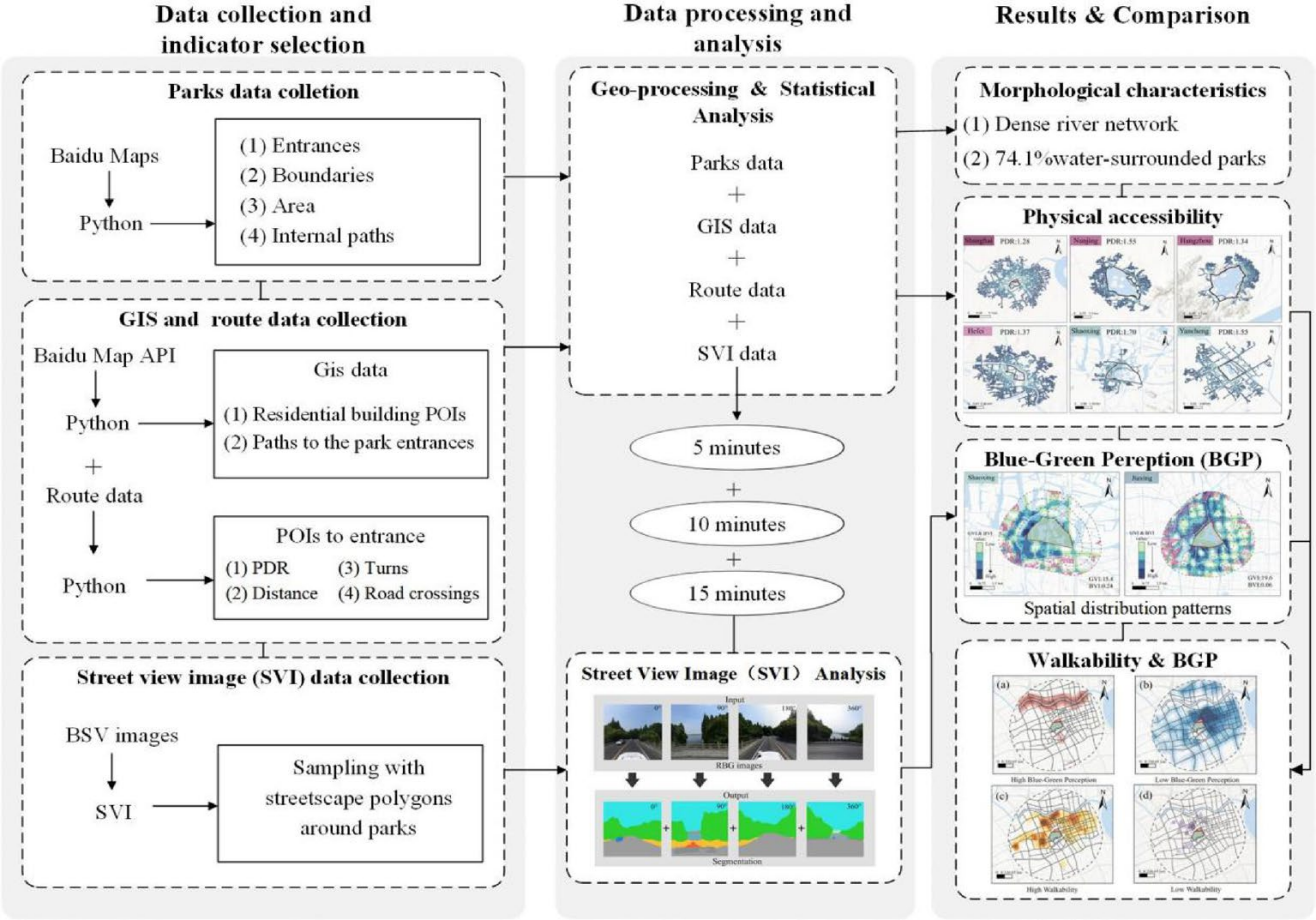
Research framework.

The research framework of this study is structured into three sequential phases (Fig. 2), each employing specialized methodologies and analytical tools. The analysis employs a hierarchical gradient of 500 m, 1,000 m, and 1,500 m, which strictly aligns with China’s *“5–10-15 min Community Life Circle”* (GB 50,180–2018) standards. This multi-threshold design enables a systematic assessment of the distance-decay effect in both physical permeability and visual perception as the service radius expands. Moreover, the experimental design targets the streetscape within a 15-min walking radius of 27 central parks. This boundary allows us to evaluate the “transit experience”—the spatial and sensory link between residential areas and park nodes. By coupling physical permeability with visual perception (GVI/BVI), we examine whether the surrounding urban fabric supports or hinders the service efficacy of the central park.

#### Geo-processing and statistical analysis

Leveraging topographic maps and high-resolution satellite imagery, we delineated the spatial extent of the 27 selected parks and identified a total of 672 formal and informal access points. A 1,500-m Euclidean buffer was established around each park boundary as a pre-filter to retrieve residential POIs via the Baidu Map API. Subsequently, actual walking routes from each POI to the nearest park entrance were generated using custom Python scripts. All spatial analyses and cartographic visualizations were executed in ArcGIS 10.8. Utilizing WorldPop 100-m resolution population data, we performed multi-scale statistical analyses encompassing individual residences, street blocks, parks, cities, and the entire YRD region. These analyses accounted for both the spatial distribution of park entrances and the surrounding urban morphology to ensure a robust assessment of service equity.

#### Street view image (SVI) analysis

We utilized Baidu Street View (BSV) to extract panoramic street-level imagery. In September 2025, images were accessed and retrieved via the Baidu Map API, ensuring synchronized acquisition times and consistent seasonal conditions across all study sites^29^. This synchronized retrieval protocol was specifically designed to maintain leaf-on consistency for vegetation, thereby eliminating potential temporal bias in visual perception metrics caused by seasonal variation. BSV provides 360° horizontal and 180° vertical coverage. The pitch (vertical viewing angle) and Field of View (FOV) were set to 22.5° and 90°, respectively, to simulate the typical human visual range^28^. Sampling points were established at 50-m intervals along the road network within 5-, 10-, and 15-min walking catchments from each park. At each sampling point, four images were captured in orthogonal directions (90°, 180°, 270°, and 360°) to ensure a comprehensive panoramic representation. A total of 40,423 images were collected, each with a resolution of 3600 × 900 pixels.

To extract blue-green elements from the 40,423 images, we utilized a Fully Convolutional Network (FCN) pre-trained on the ADE20K dataset. This model architecture has demonstrated consistent high performance in urban street-scape segmentation, with reported Mean Intersection over Union (mIoU) values exceeding 75% for vegetation and water categories in similar large-scale studies^29^. Previous validations of this FCN-based approach have shown that it can achieve a Pixel Accuracy (PA) of over 90%, providing a reliable basis for quantifying GVI and BVI across diverse urban environments. In this study, “greenery” refers to tree canopies, shrubs, and natural grass, while “water” includes perceptible rivers, canals, and ponds, ensuring the metrics strictly reflect natural blue-green infrastructure. The final visual index for each sampling site was derived by averaging the pixel proportions across the four directions, as defined below:

$${P}_{j}=\frac{\sum_{i=1}^{4}{V}_{i,j}}{4},\hspace{1em}\{i\in (\mathrm{1,2},\dots ,n)\}$$

- *P*_*j*_ represents the integrated visual perception index (e.g., average GVI or BVI) for sampling site *j*;
- *V*_*ij*_ denotes the proportion of the specific visual element (such as greenery or water) extracted from the *i*^th^ image at site *j*;
- *i* refers to the direction index of the images captured at each location (*i* = 1, 2, …, *n*);
- *j* denotes the spatial index of the sampling site;
- *n* is the total number of images captured per sampling site (in this study, *n* = 4).

#### Spatial coupling of accessibility and perception

To quantitatively integrate the dimensions of “Walking to Reach” and “Walking to See,” we employed a spatial coupling framework based on quadrant classification. Accessibility metrics (PDR, NOT, and NRC) and visual perception indices (GVI and BVI) were normalized to a 0–1 range to ensure comparability. Each sampling site was then categorized into a four-quadrant matrix to identify spatial matches and mismatches between physical network efficiency and sensory quality. Furthermore, Kernel Density Estimation (KDE) was utilized to analyze the joint spatial distribution of these two dimensions, allowing us to visualize perception “hotspots” and “deprived zones” surrounding the parks. This integrated approach effectively captures the structural and qualitative characteristics of the urban-park interface. To align with the actual navigational experiences of citizens, this study prioritizes intuitive descriptive analysis over complex mathematical modeling. By emphasizing practical legibility over technical complexity, our methodology ensures that the results are accessible, actionable, and immediately interpretable for urban practitioners and non-professional stakeholders.

## Results and analysis

### Spatial and morphological configurations

Central parks in the YRD region generally exhibit a spatial pattern characterized by being “surrounded by water systems and embedded within dense urban fabrics.” Approximately 85.2% of the sampled parks are adjacent to or encircled by water, reflecting the distinct hydrographic features of a “water-town” landscape. This waterfront characteristic constitutes a significant commonality in the morphology of regional central parks. Based on spatial scale, the 27 central parks were categorized into three types: Small-scale (0–50 ha; n = 16, 59.3%), Medium-scale (50–100 ha; n = 3, 11.1%), and Large-scale (>100 ha; n = 8, 29.6%). To rigorously address these morphological characteristics, we employed the Shape Index (SI) and the Entrance-to-Perimeter Ratio(E/P ratio) to quantify the 27 samples. The results are detailed as follows:

i. *Small-scale parks (0–50 ha)* are distributed across cities of various sizes, with an average water area of 3.9 ha (SD = 4.3) and a mean water-to-land ratio of 19.1% (SD = 17.0). Moreover, these parks demonstrate the highest variation in shape, with *SI* values ranging from 1.04 (highly compact) to 2.50 (highly irregular).This scale typically features the highest edge accessibility. These parks demonstrate the highest variation in geometric form, with a mean SI of 1.62 (SD = 0.45), ranging from 1.04 to 2.50. This morphological diversity is accompanied by high entrance density (Mean = 6.91, SD = 4.90).
ii. *Medium-scale parks (50–100 ha)* are primarily concentrated in Type II large cities and medium-sized cities, featuring an average water area of 6.56 ha (SD = 10.4) and a water-to-land ratio of 10.9% (SD = 16.9). Parks in this category shift toward more compact geometries, with *SI* values generally stabilizing between 1.10 and 1.40 (SD = 0.08). Entrance density is highestin this scale (Mean = 7.40, SD = 3.11).
iii. *Large-scale parks (*> *100 ha)* are predominantly found in megacities and certain small-to-medium cities, with the average water area reaching 202.87 ha (SD = 193.9) and the water-to-land ratio significantly increasing to 57.7% (SD = 14.9). These parks frequently exhibit the most compact forms, with a mean *SI* of 1.32 (SD = 0.38). Access points are more widely spaced (Mean = 6.40, SD = 3.49). Combined with the region’s 57.7% mean water-to-land ratio, these factors concentrate pedestrian access into specific gateways.

### Physical accessibility: “Walking to Reach”

#### Evaluating service coverage

**Fig. 3:**
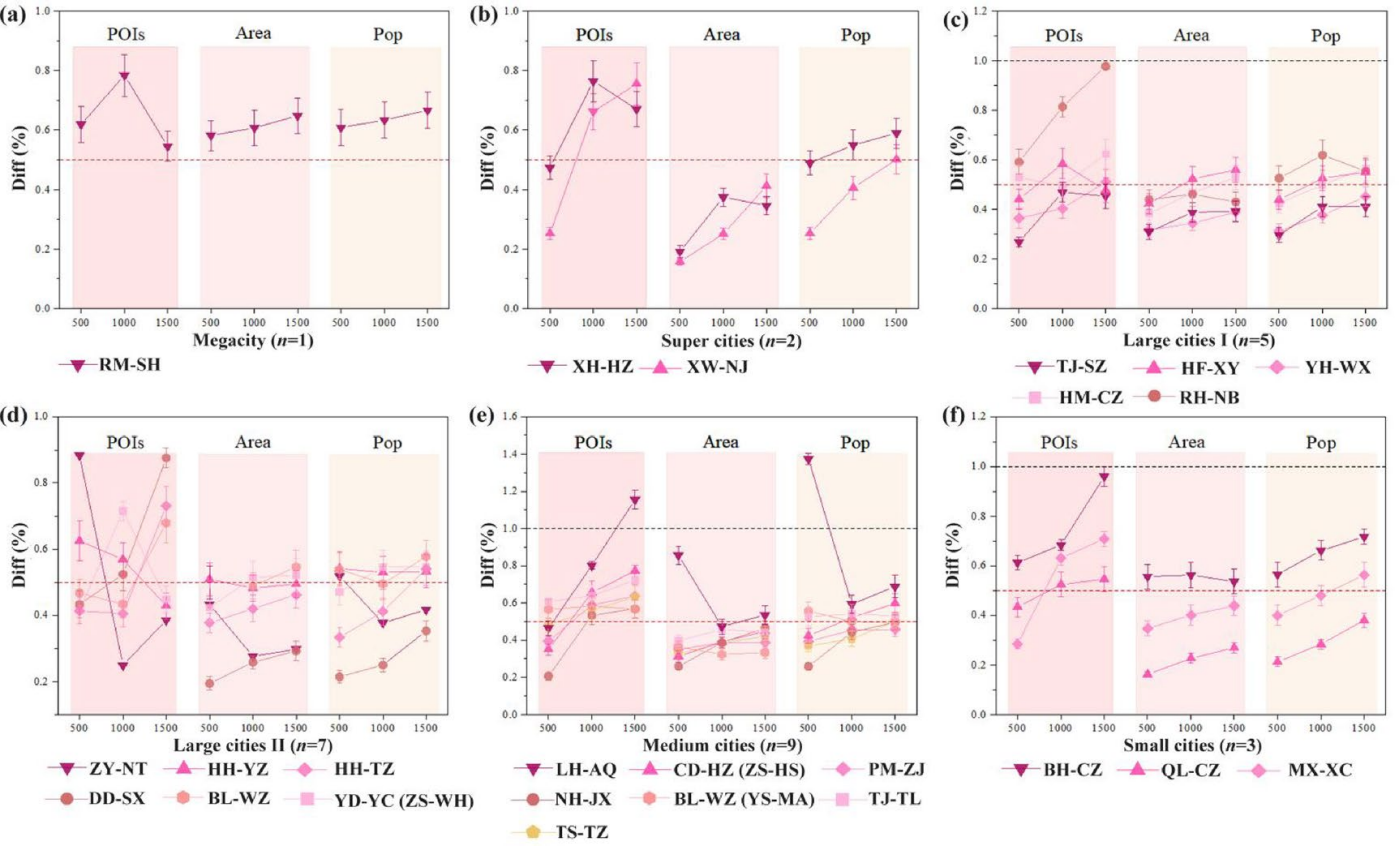
Three indicators at different distances in 27 central parks at different city scales. “Diff” represents the percentage ratio between network-based and Euclidean distances (%); (**a**)–(**f**) illustrate the trends of “Diff” for POIs, Area, and Pop across various urban scales as the distance threshold increases. Special emphasis is placed on proportions below 0.5 and above 1.0, which reflect the disparities in service levels among different cities at 5-min, 10-min, and 15-min walking intervals.

We categorized the 27 central parks by urban scale and analyzed the service accessibility of residential areas at the inter-city, intra-city, and cross-scale levels. Using network-based walking distance thresholds of 500 m, 1,000 m, and 1,500 m—which correspond to the 5-, 10-, and 15-min walking durations defined in the GB 50180–2018 standards—we evaluated the variations across different pedestrian catchments. Table 1 reveals the disparities in accessibility across urban scales, while Fig. 3 illustrates the comparative trends between individual cities. The findings indicate substantial heterogeneity in park service accessibility across different urban hierarchies, individual cities, and network-based walking ranges.

**Table 1:** Park service area in terms of various approaches, thresholds and indicators.

City scale	Park entrance	Based on Euclidean distances			Based on network walking distance		
		Residential buildings	Service radius centered on the entrance		Residential buildings	50 m service buffer around the paths	
	Distance	POIs	Area	Pop	POIs	Area	Pop
Megacity (*n* = 1)	500 m	1064	1.65	6.90	659	0.96	4.2
	1000 m	2845	4.74	18.6	2055	2.88	11.8
	1500 m	5513	9.41	35.7	3511	6.10	23.8
Super large cities (*n* = 2)	500 m	1345	12.76	9.15	470	2.27	3.45
	1000 m	3054	20.45	19.35	1684	6.25	9.3
	1500 m	5345	29.68	32.75	3325	11.17	17.9
Large cities I (*n* = 5)	500 m	579	2.27	4.42	253	0.86	1.7
	1000 m	1635	5.8	11.4	813	2.56	5.38
	1500 m	3119	10.83	21.16	1627	5.01	10.52
Large cities II (*n* = 7)	500 m	477	2.82	3.09	225	0.99	1.43
	1000 m	1297	6.58	8.24	690	2.65	3.91
	1500 m	2545	11.9	15.1	1432	5.12	7.87
Medium cities (*n* = 9)	500 m	677	3.96	3.81	315	1.89	2.8
	1000 m	1713	8.37	9.00	1030	3.46	4.58
	1500 m	2897	14.32	15.03	1916	6.31	8.78
Small cities I (*n* = 3)	500 m	352	4.18	2.87	155	1.01	1.07
	1000 m	928	9.07	7.07	513	2.88	3.3
	1500 m	1715	15.52	12.3	1084	5.56	6.7

All data represent the mean values for all cities within the same scale; “Area” denotes the service coverage area in km^2^, and “Pop” denotes the total population within the 15-min walking catchment (measured in tens of thousands), calculated via zonal statistics using the 2020 WorldPop 100-m resolution dataset.

Centered on park entrances, we calculated the number of residential building POIs, service area, and population within the 500 m, 1,000 m, and 1,500 m radii based on both Euclidean (linear) buffers and actual network-based walking paths (using a 50 m service buffer around the paths). The results demonstrate that the values for the three indicators derived from actual walking paths are only approximately 50% of those calculated using the Euclidean service radii commonly employed in traditional planning. This confirms that conventional metrics significantly overestimate actual pedestrian accessibility (Table 1). Furthermore, the disparity between urban scales tends to increase as the network walking distance decreases, suggesting that park accessibility is a non-uniformly distributed resource.

To further analyze the imbalances across urban scales, we calculated the ratio (Diff.) of network-based indicators to Euclidean-based indicators and compared the trends across different pedestrian ranges (Fig. 3). A lower Diff. value signifies a greater discrepancy between the network walking and Euclidean distances, indicating more severe physical constraints on residents’ ability to “reach” the park. Notably, the Diff. values for the network-based service area in nearly all central parks were consistently lower than those for residential POIs and population. This implies that service coverage is more acutely affected by spatial connectivity, where actual pedestrian access is heavily restricted by the configuration of the walking network. Furthermore, Large cities II exhibited significant inter-city variations in residential POIs, service area, and population indicators. These disparities within the same urban scale may stem from divergent urban development models rooted in distinct cultural and economic backgrounds (Fig. 3d). For instance, in Shaoxing, a city guided by historical and cultural preservation, the growth of service area and population 500 m to 1,000 m network distance range is relatively stagnant, resulting in lower Diff. values. Conversely, Nantong, a rapidly developing emerging city, showed marked fluctuations, particularly with a significant decline in accessibility within the 1,000 m network range. This phenomenon reflects the impact of functional land-use layouts and physical barriers—such as water bodies and arterial roads—on pedestrian accessibility.

#### Shortest routes to the central parks

**Fig. 4:**
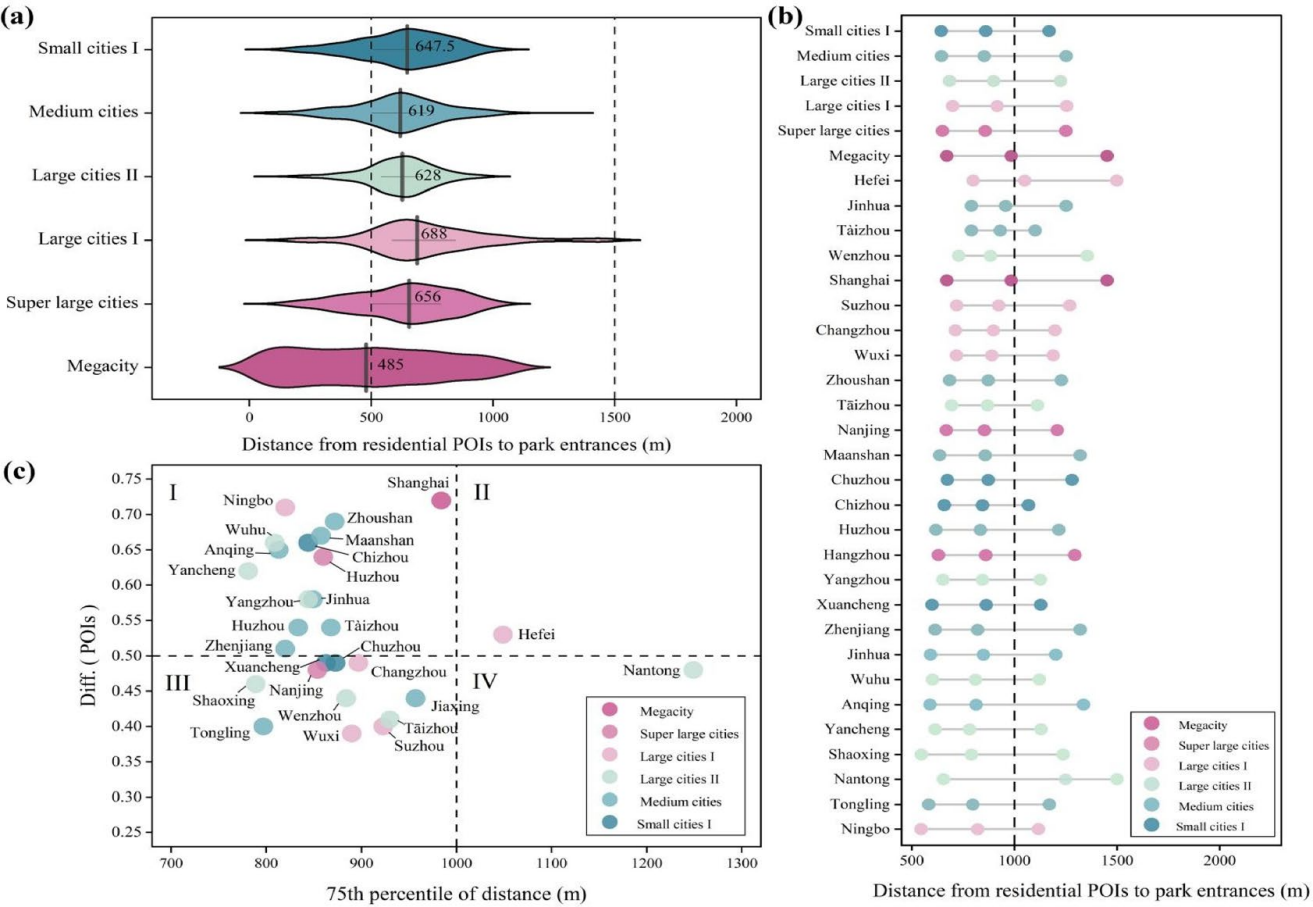
Distance from residential POIs within a 1000 m range to the nearest entrance. (**a**) Average walking distances from residential POIs to central parks within a 1,000-m catchment across different urban scales. The sample sizes (*n*) for each scale, ordered from largest to smallest, are *n* = 1, 2, 5, 7, 9, and 3. (**b**) Pedestrian access distances from residential POIs to parks. The three points for each city represent the minimum (left), mean (middle), and maximum (right) access distances within the 1,000-m catchment. (**c**) Scatter plot of the Service Disparity Index (Diff.) versus walking distance (75th percentile) for residential POIs (*n* = 27).

The morphology of shortest walking paths between residences and park entrances reflects the integration of parks into the urban fabric, directly impacting service efficiency. In this study, we analyzed shortest walking distances within a 1500 m network-based catchment, excluding routes exceeding this threshold. As shown in Table 1, Euclidean-based metrics significantly overestimate service ranges compared to network-based methods. The spatial distribution of these paths within the 1500 m network walking range (Fig. 4) reveals marked imbalances in pedestrian permeability and accessibility across different urban scales and individual cities.

According to Fig. 4a, Large cities II (SD = 238, MD = 688) and Medium cities (SD = 191, MD = 619) exhibit higher degrees of dispersion, indicating substantial disparities in residential distribution and park access distances within these urban scales. Furthermore, in all cities, over 50% of residential POIs cannot access a central park within 500 m network distance, and nearly 25% fail to reach one within 10 min (Fig. 4b). While 24 cities in the YRD can cover a large proportion of residents within a 1000 m network distance, varying degrees of disparity in pedestrian permeability remain.

To further investigate the equity of park accessibility across different cities and scales, we constructed a framework based on the Service Disparity Index (Diff.) and actual network walking distance. The walking distance was measured at the 75th percentile, representing the path length required for the majority of residents (75%) to reach the nearest park entrance. Using Diff = 0.5 and Distance = 1,000 m as benchmarks, the 27 sample cities were categorized into four quadrants: (I) High Coverage-Short Distance, (II) High Coverage-Long Distance, (III) Low Coverage-Short Distance, and (IV) Low Coverage-Long Distance (Fig. 4c).

The majority of central parks (n = 25) are distributed in Type I (n = 14) and Type III (n = 11) (Fig. 4c). Notably, 57.1% of Type I parks are located in small and medium-sized cities (e.g., Anqing, Huzhou), suggesting that under conditions of lower construction intensity and relatively abundant land resources, these cities more easily achieve dual optimization of service coverage and accessibility. Conversely, 75% of large cities I are categorized as Type III (Fig. 4c); despite having shorter walking distances, they exhibit lower service coverage, reflecting a mismatch between green space layout and service demand in high-density built-up areas. It is noteworthy that Hefei (Large city I) and Nantong (Large city II) correspond to Type II and Type IV (Fig. 4c), respectively, deviating from the overall trends of their peers, likely due to their specific green space structures and transport access models. These findings suggest that walking distance is not the primary limiting factor for park access; rather, the significant disparities in coverage—driven by variations in pedestrian permeability—warrant greater attention.

#### Pedestrian permeability and route complexity

**Fig. 5:**
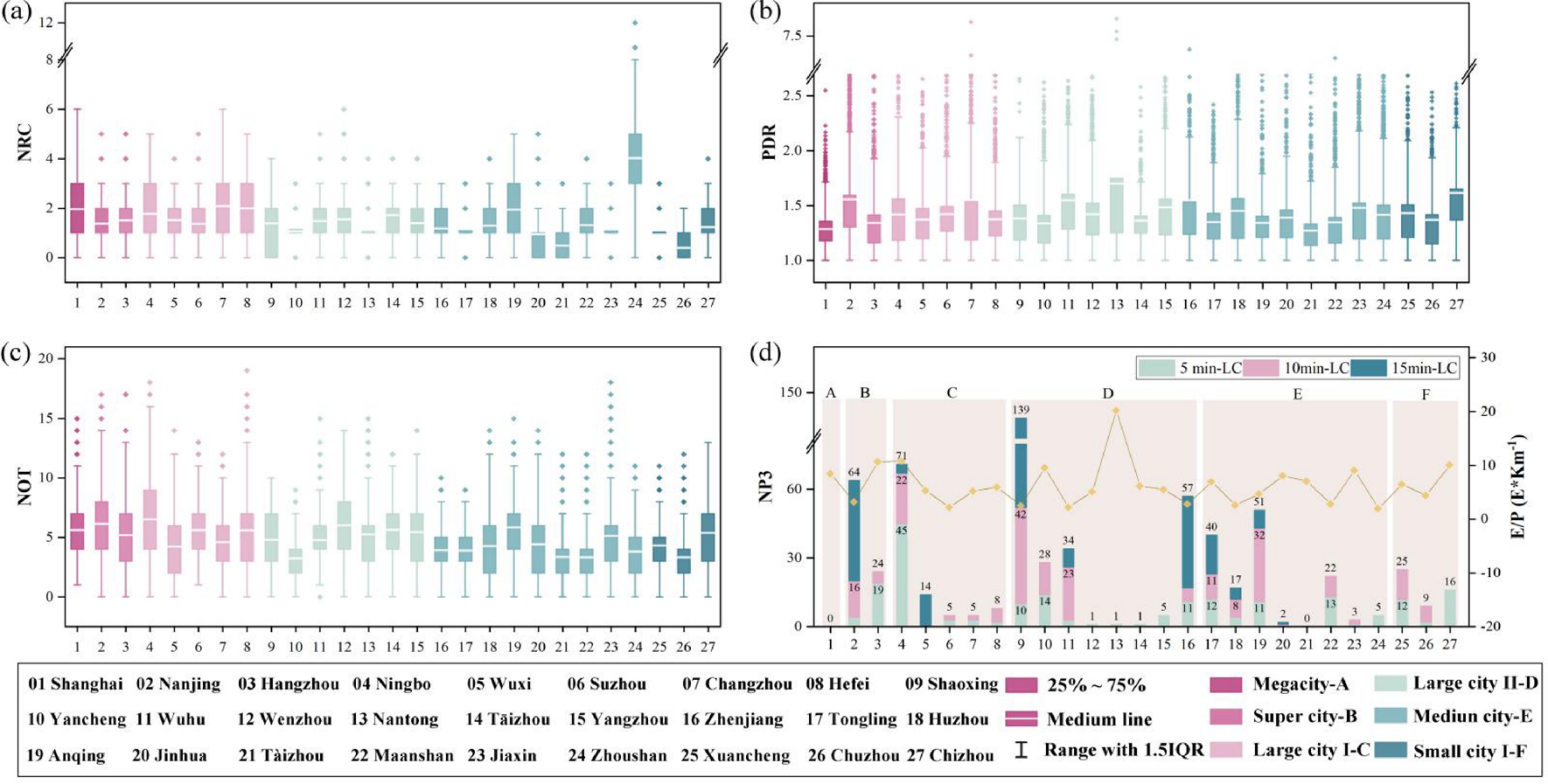
Pedestrian permeability metrics within a 15-min catchment for central parks in the Yangtze River Delta. The median is used to reduce bias due to significant variations in population or building area at each POI. (**a**) Number of road crossings (NRC) within a 15-min walking range; (**b**) PDR within a 15-min walking range; (**c**) Number of turns (NOT) within a 15-min walking range; (**d**) High-circuity paths (PDR > 3.0, NP3) and corresponding entrance-to-perimeter (E/P) ratios within a 15-min walking range. NP3 refers to the number of paths with PDR > 3.0.

Pedestrian permeability was evaluated using the pedestrian directness ratio (PDR), number of turns (NOT), and number of road crossings (NRC). The PDR (the ratio of network path distance to Euclidean distance) serves as a key indicator of route directness and urban connectivity. Following established benchmarks in urban morphology^36^, where a PDR close to 1.0 represents perfect directness and values significantly higher indicate increasing structural impedance, we defined a PDR ≤ 1.3 as representing high permeability^37,38^, while a PDR > 3.0 was set as the threshold for poor permeability^39^. This cutoff specifically identifies areas of extreme circuity—where walking distances are more than three times the linear distance—often resulting from significant physical barriers such as water bodies or fragmented street networks We analyzed these metrics within 1500 m network walking distances to assess the spatial connectivity between central parks and the surrounding urban fabric (Fig. 5).

**Fig. 6:**
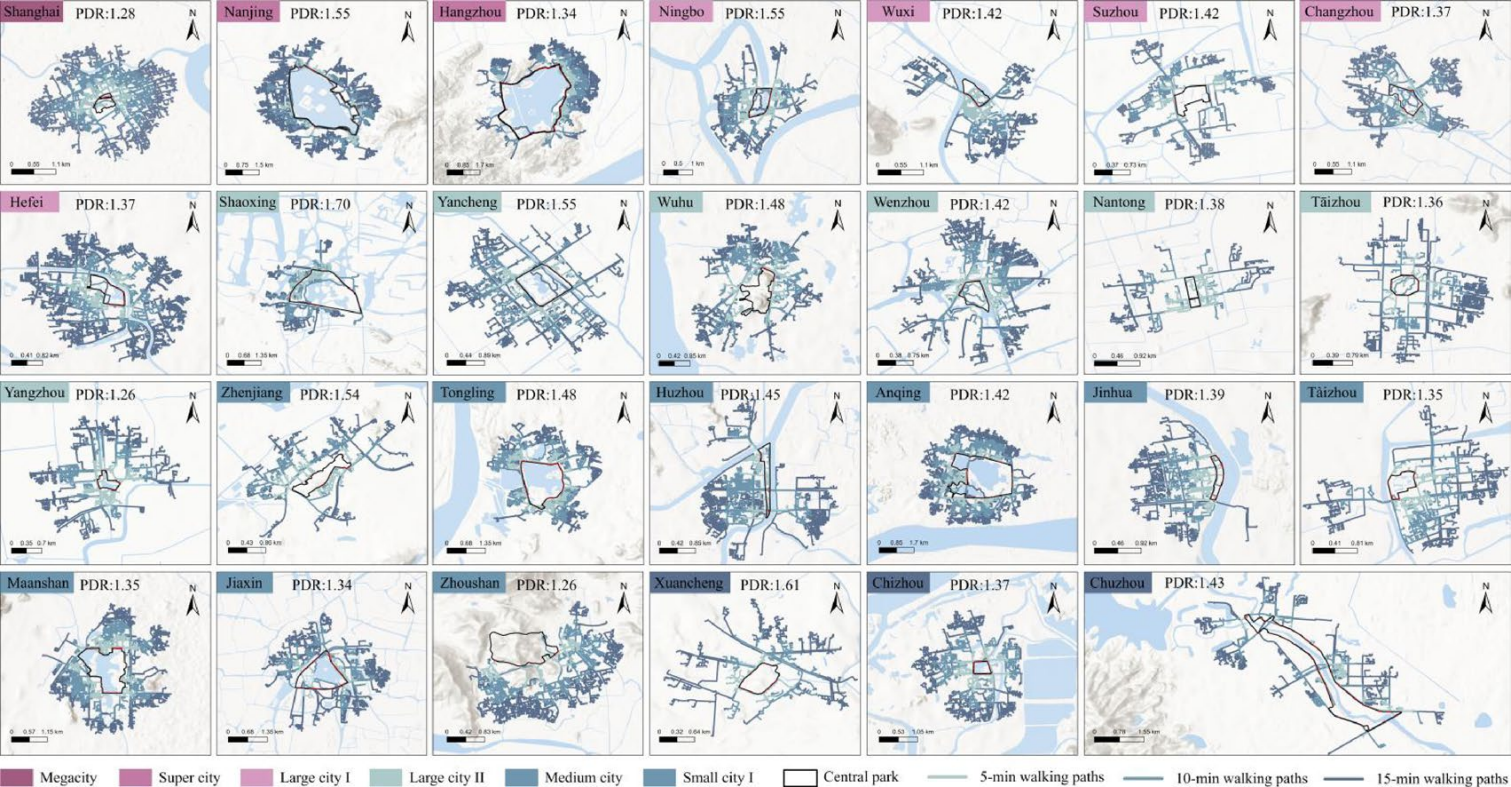
Spatial distribution of high-permeability walking paths (PDR ≤ 1.3) within 5-, 10-, and 15-min life circles across 27 central parks in the Yangtze River Delta.

The average PDR in the YRD is 1.43 (median: 1.33). Among 647 routes with PDR > 3.0 (NP3), approximately 70% required more than 5 min of walking, highlighting significant access barriers (Fig. 5d). Overall, 37% of YRD central parks exhibit pronounced residential access disadvantages. These high-PDR residential POIs are primarily concentrated in Type I and II large cities and medium-sized cities with complex street networks (Figs. 5 and 6). Notably, only Shanghai and Zhoushan maintained average PDR values below 1.3. In the case of Zhousha, this higher walking efficiency is reflected in elevated NRC values (Fig. 5a) and NOT values (Fig. 5c), while Shanghai’s performance indicates a more integrated network structure despite its high-density environment.

To investigate the underlying causes of poor permeability, we employed the entrance-to-perimeter (E/P) ratio, defined as the number of validated entrances per kilometer of the park boundary (entrances/km). This metric serves as a direct measure of edge porosity, where a lower ratio indicates fewer access opportunities and necessitates longer detours along the park’s exterior. Our analysis of the five parks with the highest PDR values provides quantitative evidence that low permeability is driven by both a low frequency of access points and their uneven spatial distribution (SD = 375.2). These physical barriers—often manifested as fences or restricted management—force pedestrians to bypass large segments of the park perimeter, thereby significantly increasing path costs and access inequality (Table 2). The statistical linkage between a low E/P ratio and observed PDR values confirms that even in dense urban fabrics, the insufficient ‘porosity’ of the park edges remains a decisive constraint on urban-park integration.

**Table 2:** Inter-entrance distances for high-circuity paths (PDR > 3.0) from residential POIs to park entrances (ranked by PDR in descending order).

City scale	City name	PDR (Ave)	E/P ratio (km^−1^)	Inter-entrance distances				
				Max	Min	Ave	MD	SD
Large city II	Shaoxing	1.7	2.5	1962	58	396.8	271	449.61
Small city I	Xuancheng	1.61	6.5	411	55	154.6	106	108.47
Super city	Nanjing	1.55	3.2	1453	12	316.13	125	406.78
Medium city	Zhenjiang	1.54	2.8	1264	19	357.8	176.5	412.59
Large city II	Wuhu	1.48	2.2	1505	40	461.2	134	498.7

### Visual perception: “Walking to See”

#### Visual characteristics of blue-green spaces

Regarding green space perception, the average GVI surrounding central parks in super-large cities is 22.75% (SD = 15.4). Across the walking catchments of all 27 sampled parks in the YRD, only 22% of the sampling sites achieved a GVI > 25%, with a minority of 8.5% exceeding 35% (Table 3). In contrast, 55% of the surveyed areas fell below a 15% GVI threshold. These figures highlight a internal disparity in green perception within the pedestrian catchments.

**Table 3:** Visual perception metrics of blue-green spaces across different urban scales.

City Scale	GVI (%)			BVI (%)			GVI (1500 m network walking paths) (%)					B_0_ (%)	B_0_ & G_25_ (%)
	Max	CD	SD	Max	CD	SD	≤ 5	5–15	15–25	25–35	> 35		
Megacity (*n* = 1)	57.4	10.1	10.5	9.2	0.03	0.40	41.5	28.3	18.9	8.2	2.7	17.1	1.8
Super-large cities (*n* = 2)	71.4	22.8	15.4	6.7	0.07	0.48	15.1	24.5	19.1	17.8	23.6	22.3	8.9
Large cities I (*n* = 5)	56.1	13.8	11.2	11.8	0.12	0.84	26.6	35.8	21.6	10.7	5.4	42.7	10.1
Large cities II (*n* = 7)	54.6	14.6	10.5	11.6	0.09	0.63	22.4	32.2	24.9	10.5	4.7	40.8	10.2
Medium cities (*n* = 9)	56.4	16.0	10.8	8.4	0.06	0.50	18.9	33.4	26.1	14.9	6.6	32.9	15.4
Small cities I (*n* = 3)	55.1	17.3	11.1	10.2	0.08	0.48	16.0	35.4	32.8	18.1	8.0	25.3	15.1

G_25_: Represents GVI ≥ 25% (high-quality green perception); B_0_: Represents BVI > 0 (visible blue space); B_0_ & G_25_: the percentage of sampling sites that simultaneously achieve high-quality green perception and visible blue space.Max refers to the maximum value; SD refers to the Standard Deviation; CD refers to the Coefficient of Variation (calculated as *SD/Mean*), providing a normalized measure of probability distribution for GVI and BVI across different sample sites.

In terms of blue space, we evaluated BVI within 20-m buffers—a distance threshold identified as effective for blue space perception^46^. Results indicate that while the average proportion of visible blue space (B0, representing BVI > 0) in megacities is 17.1% (lower than in small and medium cities), a trend that contrasts with the green space pattern and likely stems from the unique natural hydrological structures of the “Jiangnan water-towns. However, 62.8% (SD = 14.4) of sampling sites within the 20-m “watershed” failed to identify blue space, illustrating the “proximal but invisible” phenomenon. Furthermore, an average of 26.2% (SD = 12.2) of blue space areas fail to achieve high-quality green perception (GVI < 25%). A comparative analysis of GVI and BVI distributions suggests no apparent linear relationship between the two at the visual level, suggesting that water bodies and green spaces are spatially independent, often resulting in “greenery without water” or “water with sparse greenery.”

**Fig. 7:**
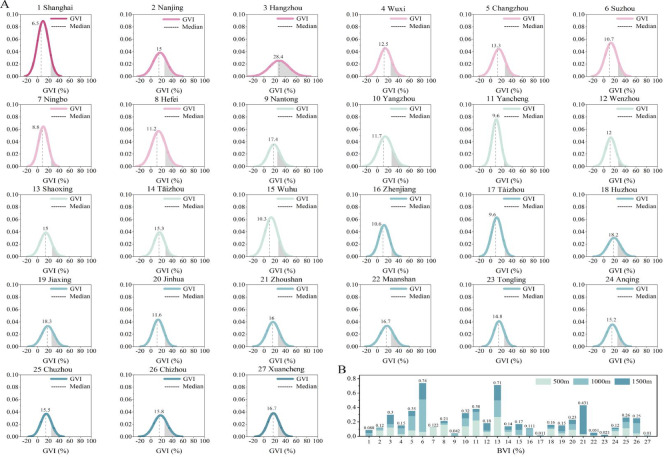
Distribution characteristics of GVI and BVI across 27 central parks in the Yangtze River Delta. (**A**) Normal distribution curves and median values of GVI for each city. The shaded grey areas represent the proportion of sampling sites where GVI ≥ 25% (high-quality green perception); (**B**) Variations in average BVI values across different walking thresholds for the 27 sampled cities.

**Fig. 8:**
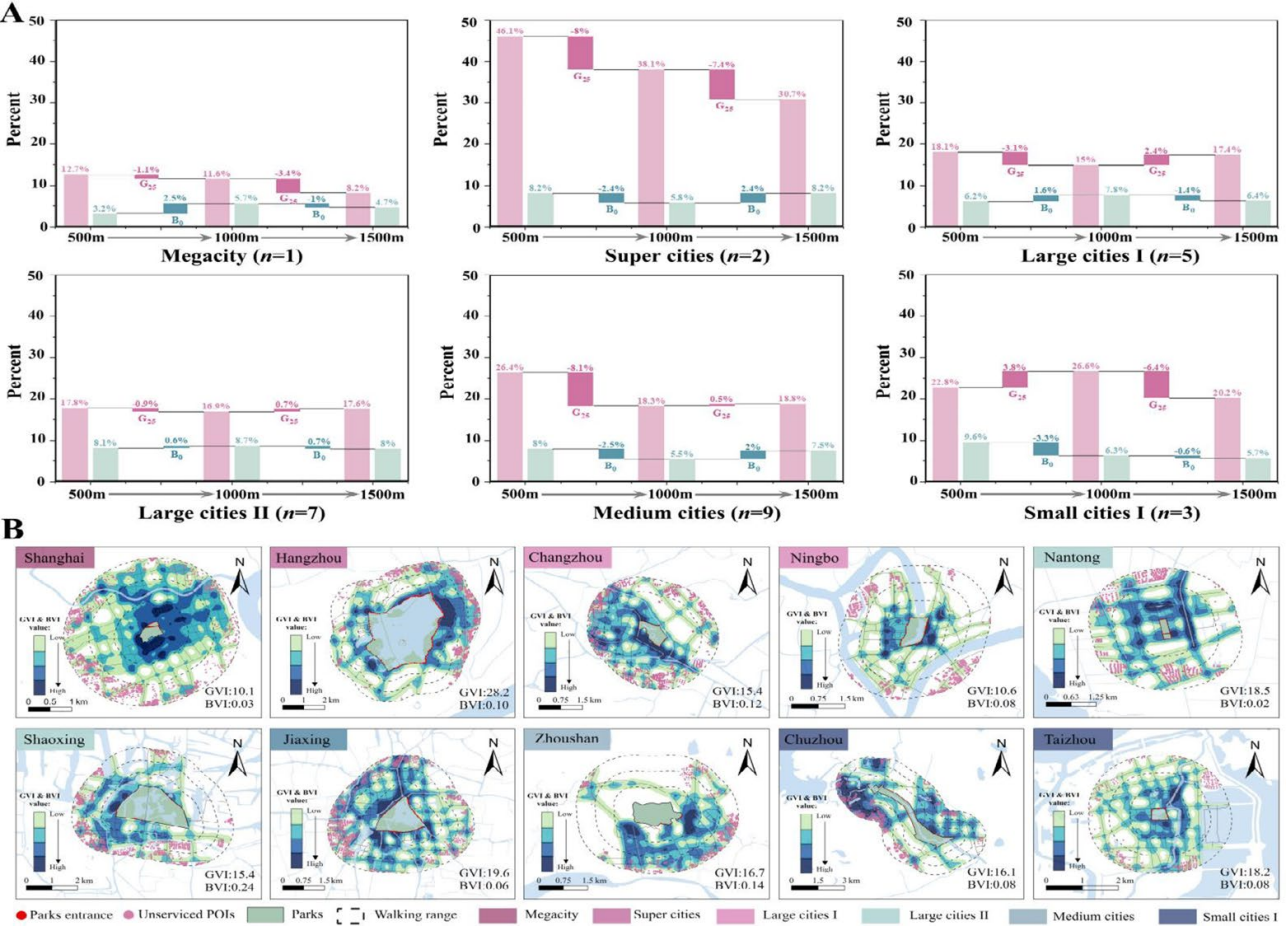
Variation trends and spatial distribution patterns of GVI > 25% and BVI > 0 across different urban scales within 5-, 10-, and 15-min walking catchments. (**A**) Overall variations of GVI and BVI across three walking catchments for six urban scales. The bridge (or step) charts illustrate the fluctuations, where red indicates changes in GVI and blue indicates changes in BVI, (**B**) Kernel Density Estimation (KDE) of blue-green perception and distribution of unserved residential POIs for 10 representative cities across six urban scales.

Analysis of GVI and BVI distribution characteristics across the 1,500 m network-based walking catchments (Figs. 7 and 8) reveals that sensory disparities tend to decrease as urban scale diminishes. For GVI distribution (Fig. 8A), with the exception of Hangzhou (M = 28.4%), the median GVI in most cities is below 25%, indicating limited overall visual exposure to green space. Although large cities (e.g., Shanghai, Nanjing, Ningbo) exhibit higher concentration in their distribution curves compared to smaller cities, their overall medians and high-GVI (> 25%) spatial distributions are relatively lower. In contrast, smaller cities show more dispersed curves, reflecting a “polarized” green perception where advantageous areas are highly concentrated while disadvantaged areas lack sensory exposure.

Regarding BVI spatial distribution, we analyzed the mean BVI fluctuations across 500 m, 1000 m, and 1500 m network walking scales (Fig. 8B). Blue perception fluctuates considerably more between cities than green perception, with the most pronounced disparities occurring within the 1000 m range. Certain cities (e.g., Wenzhou, Huzhou, Jiaxing) exhibit peak BVI values at the 1000 m threshold, highlighting superior water visibility at medium spatial scales. Conversely, cities such as Wuxi and Yangzhou show overall lower BVI trends. Blue space exposure appears highly city-dependent, closely tied to specific local hydrological characteristics (Fig. 7).

#### Blue–Green perception and spatial patterns

By analyzing blue-green perception across the three walking catchments, we observe that both the high blue-green perception (G25 and B0) and the distribution patterns weaken as the distance from central parks increases This trend differs from the patterns of pedestrian permeability. In super-large cities, although high green perception (G25) declines markedly with distance. However, the overall blue-green perception remains higher than in other urban scales (Fig. 8A(b)). This shows that the blue-green perception of megacity central parks is largely limited to a 500 m radius. Specifically, high GVI is found only near the parks, while GVI in the 1000 m-1500 m range is poor. This indicates a lack of visual connectivity between these parks and the wider urban environment.

In contrast, large cities II show a stable but low-level distribution. Both G25 and B0 metrics showing minimal change across all three scales (Fig. 8A(d)). Large cities I display a “V-shaped” curve, with high GVI in the center and abundant blue space in the periphery (Fig. 8A(c)). For medium and small cities, high green perception (G25) is relatively high within the 500 m radius but drops sharply at greater distances. In small cities, B0 also decreases at the 1500 m scale (Fig. 8A(e–f)). Further analysis reveals that residents in areas lacking physical access also suffer from low blue-green perception. By coupling GVI and BVI data from all sampling sites via kernel density estimation (KDE), we identified the distribution patterns of blue-green perception surrounding the parks (Fig. 8B).

High blue-green perception hotspots are mostly located within 500 m of parks or near major entrances, particularly in large and super-large cities. This indicates that blue-green perception in urban planning highly depends on the layout of park entrances and pedestrian corridors, especially those near transit hubs and dense residential areas. In cities like Shanghai and Hangzhou, these hotspots align with primary transportation nodes (Fig. 8B). Conversely, areas with low blue-green perception usually appear in the 1000 m-1500 m range. These areas are both physically remote but also perceptually isolated due to broken green connectivity. Interestingly, these areas often cluster near water bodies that are physically present but hidden from view. While some areas with high GVI values, their perceptual benefit is restricted by poor visibility and fragmented paths. In contrast, high blue-green perception zones tend to form continuous corridors along accessible water bodies (Fig. 8B).

## Discussion

### Spatial mismatch between “Walking to Reach” and “Walking to See”

**Fig. 9:**
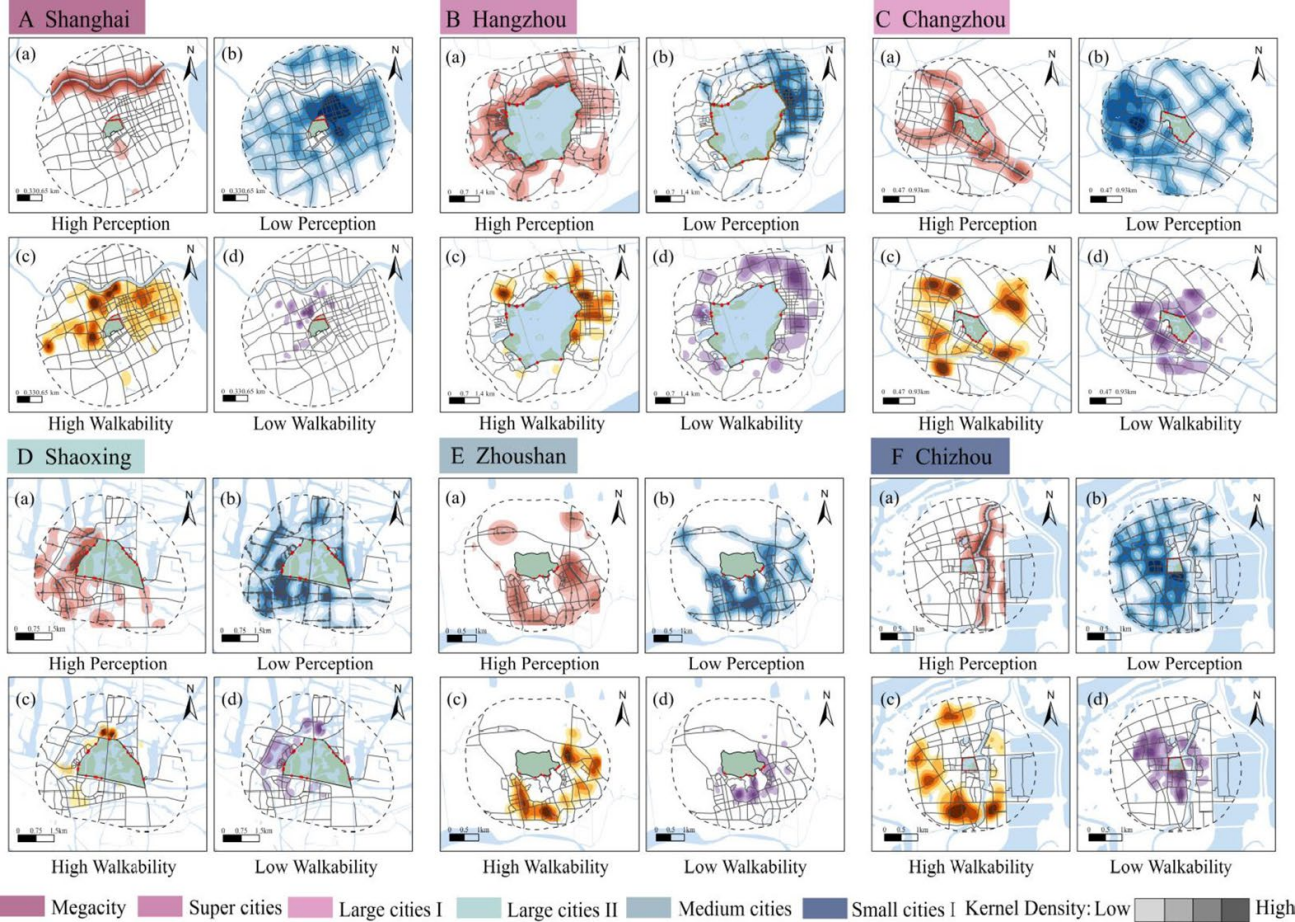
Spatial distribution patterns of blue-green perception (GVI & BVI) and walkability (based on residential POIs) for six representative central parks across different urban scales.

As central landmarks of urban identity and livability, city parks are intended to ensure equitable resource distribution. However, their benefits are often spatially restricted. This study reveals that the population and area effectively served within 1500 m network walking distances are substantially smaller than those estimated by traditional Euclidean buffers. As the walking radius expands, walkability and blue-green perception exhibit divergent trends. This inequality is reflected not only in quantitative metrics but also in spatial patterns influenced by urban scale and environmental context. To avoid the subjectivity inherent in multi-criteria weighting, this study evaluates the coupling of ‘See’ and ‘Reach’ dimensions through layered spatial overlay. This approach preserves the high-resolution granularity of both visual and physical datasets, enabling a more direct identification of areas where high sensory potential is fragmented by physical barriers. In this framework, ‘High Walkability’ is primarily defined by a PDR ≤ 1.3, representing optimal pedestrian permeability, while ‘Low Walkability’ corresponds to PDR > 3.0, indicating severe physical fragmentation. Regarding visual perception, ‘High Perception’ requires the simultaneous fulfillment of GVI > 25% and BVI > 0. Conversely, ‘Low Perception’ is defined as sites where either GVI ≤ 25% or BVI = 0. By applying these consistent thresholds across 27 cities, we categorized the urban spaces into four distinct spatial models (Table 4 and Fig. 9).

**Table 4:** Four spatial distribution models and their underlying influential factors.

Types of spatial patterns		Spatial distribution	Influencing factors	Typical cities
A	High perception and high walkability	On both sides or at the confluence of rivers and lakes	–	Tāizhou, Suzhou, Hangzhou
B	High perception and low walkability	Concentrated in areas with dense water-land interfaces near parks or along the foothills	Gated community barriers	Shaoxing, Ningbo, Yancheng, Anqing, Chizhou
			Historic structures barriers	
			Urban arterial road barriers	
			Natural barriers (water & terrain)	
C	Low perception and high walkability	Areas 1000–1500 m around parks, characterized by dense street networks but not adjacent to water	Road widening	Shanghai, Yangzhou, Changzhou
			Overpasses and retaining walls	
			Retreat of vegetation planting	
D	Low perception and low walkability	Park-adjacent areas with dense arterial roads but lacking direct water access	All of the above factors	Zhenjiang, Tongling
			Park closure management	
			Absence or discontinuity of entrances

A prevalent phenomenon is that High-Perception/High-Walkability (HP-HW) zones are predominantly clustered along water systems or network intersections (Fig. 9). This aligns with research suggesting that blue spaces near residential areas may be more readily identified and perceived. Among all urban scales, small and medium-sized cities (SMCs) demonstrate the strongest blue-green perception (CD = 15.3%), which could be attributed to a higher synergy between natural landscapes and urban form. Cities like Chizhou appear to preserve intact hydrological patterns by integrating urban development with rivers and lakes (Fig. 9F). In contrast, Shanghai as a megacity, exhibits the lowest perception levels (CD = 1.8%). This case-specific disparity points to a potential “functional-sensory decoupling” possibly associated with its advanced development stage: as a dense megacity, Shanghai’s high-density infill and prioritization of transportation efficiency may lead to the “visual compression” of blue-green corridors. Notably, walkability within a 5-min radius of parks is often inferior to that of the 10–15 min environment, diverging from the expectation that proximity to green space inherently improves walkability (Fig. 9). Street-view analysis suggests this might be caused by physical barriers, as well as arterial roads and gated communities. Natural obstacles like water bodies also play a role (Table 4). This pattern resonates with multi-city accessibility assessments^20^, which highlight the challenges of infrastructure permeability in dense urban cores. Our exploration further suggests that areas with high road connectivity may emerge as impedance islands. In these zones, park resources are physically adjacent but functionally or perceptually segregated by gray infrastructure, potentially due to prioritized high-speed transit corridors that fragment fine-grained pedestrian networks. Additionally, legacy land-use boundaries likely restrict direct park access.

Furthermore, the interaction between blue-green perception and walkability creates a distinct “Perception-Walkability Mismatch.” In the specific case of Shanghai, high spatial connectivity does not necessarily translate into high perception (CD = 7.75%). This discrepancy may be linked to the historical evolution of urban infrastructure, where a structural misalignment often exists between modern transit networks and legacy ecological footprints. While new arterial roads prioritize regional connectivity, they may bypass or wall off older park systems, potentially leaving these green spaces functionally marginalized. Consequently, these parks may shrink into isolated landscape fragments—visually pleasing but poorly integrated into the surrounding street fabric. In cities like Shanghai, Hangzhou, and Changzhou, low-perception areas cluster around arterial road networks (Fig. 9). Street-view data shows that poor blue perception stems from riverbank obstructions or fencing, while low green perception may be exacerbated by a lack of vertical vegetation layers or restricted sightlines (Table 4). While many studies focus primarily on green space^9^, our data highlights the critical role of blue spaces in the YRD. Riverbank obstructions often contribute to a visual-physical gap in historical districts. Conversely, SMCs tend to offer more continuous blue-green structures, potentially fostering stronger visual and emotional connections to nature. However, in cities like Huzhou and Chizhou, natural barriers (e.g., water bodies and slopes) can enhance blue-green perception while simultaneously fracturing the pedestrian network. This planning legacy suggests a tendency to prioritize viewing over access, treating parks as distant visual anchors rather than permeable urban nodes.

Regardless of urban scale, every city contains perception-walkability blind spots. These zones typically occur at park boundaries that lack entrances or directional systems (Table 4). These blind spots may reflect the impact of historical policy interventions, specifically the institutional fragmentation between blue and green infrastructure management. A lack of departmental coordination can result in the spatial independence of green–blue perception. Paradoxically, these blind spots frequently overlap with areas theoretically considered most attractive, such as waterfronts or large park edges. Our results reveal a sharp spatial contrast (Fig. 9): areas categorized in planning theory as “high-potential” zones—typically characterized by extensive water-green interfaces—do not always translate into actual experiential efficacy^26,40^. This misalignment suggests that theoretical attractiveness may fails to translate into functional permeability due to the aforementioned structural barriers.

### Policy implications: Multi-scale strategies for reconciling reach and see

With its robust ecological foundation, the Yangtze River Delta (YRD) urban agglomeration possesses unique potential to develop “Park City” morphologies that integrate blue and green infrastructures. This potential aligns with both existing water networks and highly integrated regional transport and economic systems^25^. The identified spatial inequities in walkability and blue-green perception across different urban scales highlight the need for multi-scale planning interventions to harmonize park resource distribution.

#### Regional scale: scale-dependent planning

At the regional level, spatial observations suggest that intervention policies could prioritize central parks as benchmarks for ecological civilization. Located in high-density urban cores, these parks serve as vital structural nodes—integrating ecological, recreational, and social functions^19^. In response to the identified spatial heterogeneities, planning frameworks may benefit from being tailored to urban scales:

Megacities and Super-large Cities These cities often face a “High Reach–Low See” paradox. Central parks are typically situated in highly commercialized, mature districts where rapid urbanization has led to the “decentralization” of green space supply—pushing new developments to the periphery, similar to patterns in London or New York^1,41^. Since large-scale land acquisition is often restricted in the core, our analysis suggests that strategic focus could shift from expanding greenery to reconfiguring the existing park-street interface. To address the identified perception-accessibility mismatch, morphological interventions might prioritize enhancing the openness of existing greenery and expanding visual corridors^42,43^. By optimizing boundary permeability—such as by increasing entrance density and removing opaque physical barriers—planners could ensure the continuity of sensory experiences within the 15-min walking catchment.

Large Cities I and II These cities exhibit two structural archetypes. Rapidly expanding “new town” models mirror the challenges of megacities and could be candidates for pocket-greenery interventions. Conversely, historical districts often feature high visual identity but demonstrate poor walkability due to fragmented street networks or natural barriers^44,45^. To address these identified disparities, planning could consider prioritizing micro-scale street connectivity and optimized pedestrian crossings to potentially bridge the reach-see gap.

Small and Medium-Sized Cities (SMCs) SMCs are often characterized by a “High See–Low Reach” characteristic, where natural topographies provide strong blue-green perception but coincide with restricted physical movement. Unlike Western SMCs, where poor walkability often stems from a lack of infrastructure^27^, the barriers here appear primarily structural and natural. For resource-based cities like Huzhou, Chizhou, and Xuancheng (approx. 33.3% of the sample), our spatial evidence implies that central parks could effectively act as visual anchors. Planning might aim to reinforce local identity by aligning sightlines with natural landmarks to enhance spatial legibility^46^.

#### Micro scale: reconciling the perception-accessibility mismatch

To address localized inequities, micro-scale adjustments informed by environmental factors could be explored (Table 4)

Enhancing visibility (low see / high reach) In areas where blue space is abundant but green perception is lacking, the removal of street obstacles could potentially improve sightlines. For visual gaps associated with wide roads or topographical shifts, the integration of vertical greening and landscaped medians may contribute to a more immersive pedestrian environment.

Improving transparency (high reach / obstructed see) In green-dense areas where blue spaces are hidden, replacing solid walls with permeable fencing presents a potential solution. Regulating riparian vegetation could further foster “proximity and intimacy” of water bodies. Urban designs that prioritize the preservation of view corridors may prevent infrastructure from becoming sensory barriers.

Overcoming barriers (high see / low reach) Where perception is strong but permeability is low, spatial optimization could focus on addressing physical and natural obstacles. For physical barriers (e.g., arterial roads, gated walls), options such as adding community gates, pedestrian bridges, and park entrances could be considered. For natural barriers (e.g., rivers, slopes), emphasis might be placed on waterfront boardwalks and pedestrian bridges that link disconnected neighborhoods to parks. Integrating these into a “slow-travel” network offers a potential pathway to mitigate spatial fragmentation and elevate the overall walking experience.

### Limitations and future research

Despite its contributions, this study has several limitations. First, while street-view data was sampled at 50-m intervals, coverage gaps remain in gated communities and pedestrian-only zones. Additionally, the reliance on Baidu Street View introduces potential temporal and seasonal biases, as imagery captured at different times may not fully represent the year-round state of blue-green infrastructure. Future research should integrate high-resolution satellite imagery to ensure more comprehensive spatial analysis. Furthermore, the inherent urban hierarchy of the YRD region results in an unbalanced sample distribution across city tiers, particularly with Shanghai serving as a singular representative of the megacity scale. Therefore, future studies could extend beyond the YRD region to include a more balanced and diverse sample of cities across different tiers. Second, our model primarily considers residential-to-park paths, potentially overlooking daily trips from workplaces or transit hubs. Incorporating diverse user trajectories would yield a more holistic service range. Additionally, while we focus on the statutory 15-min walking sphere, future research should explore broader service boundaries for single-city cases to account for inter-regional leisure trips that exceed daily pedestrian scales.

Moreover, accessibility and perception are subjective and vary by demographic groups. While this study uses a standardized walking baseline to ensure methodological consistency across 27 cities, this macro-scale approach primarily represents the general adult population and assumes a homogeneous walking ability. We acknowledge that vulnerable groups, such as the elderly and children, may exhibit different walking capacities and perceptual sensitivities. Future research could further refine these findings by incorporating age-stratified mobility parameters to provide more nuanced insights into the diverse lived experiences of different urban residents.

Finally, the reliance on open-source data precludes the assessment of micro-scale or transient factors, such as pavement quality and temporary obstructions. Moreover, while GVI and BVI are robust quantitative indicators, they serve as optical proxies for visual exposure and cannot fully capture the multi-sensory or subjective nature of human perception. Integrating real-time sensing, social media sentiment analysis, or field audits would further refine the evaluation of the pedestrian experience. While previous studies have demonstrated that pre-trained FCN models maintain high relative consistency across diverse urban environments, we acknowledge the lack of local fine-tuning for the FCN model as a methodological limitation. Potential domain gaps between the ADE20K dataset and the specific urban context of Chinese cities might affect the absolute precision of GVI/BVI measurements.

## Conclusions

As a globally significant megacity region, the Yangtze River Delta (YRD) provides a critical lens for examining urban livability by integrating pedestrian potential with visual perception. By analyzing central parks across 27 YRD cities, this study integrates visual perception and physical accessibility into a unified evaluation framework. The research identifies a pervasive “perception-accessibility mismatch,” revealing that certain natural areas remain difficult to access, while the most accessible paths often lack high-quality natural features. This suggests that the utility of urban parks depends on the balance between “Walking to See” and “Walking to Reach,” rather than solely on the walking distance emphasized in traditional models, which often ignore visual appreciation.

The results demonstrate that park accessibility varies markedly across urban scales, with spatial inequality becoming more pronounced at shorter distance thresholds. Notably, approximately 37% of the sampled YRD cities exhibit a comparative disadvantage in park equity, with over half of the analyzed paths characterized by low permeability. Furthermore, blue-green perception is primarily concentrated along water networks, yet distribution patterns are highly scale-dependent: large cities generally exhibit superior green perception (characterized by higher G15 proportions) while smaller cities demonstrate stronger blue perception (evidenced by greater B0 coverage). Although large cities generally exhibit superior green perception and smaller cities demonstrate stronger blue perception, areas theoretically considered “ideal” for pedestrian activity under conventional planning models may fail to deliver actual experiential effects. This discrepancy highlights that theoretical attractiveness—often assumed by planners to be inherent in blue-green assets—does not naturally translate into functional accessibility without intentional, street-level integration.

The practical value of this study lies in providing a diagnostic framework for targeted urban renewal. Our findings suggest that policymakers could implement low-cost, high-impact micro-interventions tailored to specific mismatches. For instance, in areas with high visibility but low accessibility, intervention strategies might emphasize “breaking barriers” by adding community gates or pedestrian bridges. Conversely, for locations that are highly accessible but offer poor visual experiences, efforts could focus on “sensory enrichment” through vertical greening and the removal of street obstacles. Such an approach may enable urban managers to allocate budgets more efficiently, supporting the development of urban park systems. Ultimately, this research offers a new governance model for megacity regions facing land constraints. It demonstrates that achieving park equity requires integrated attention to both sensory experience and physical accessibility.

## Data Availability

The datasets used and/or analysed during the current study **are** available from the corresponding author on reasonable request.

## References

[1] Pearsall, H. & Eller, J. K. Locating the green space paradox: A study of gentrification and public green space accessibility in Philadelphia, Pennsylvania. *Landsc. Urban Plan.* **195**, 103708 (2020).

[2] Qin, X., Wei, Y. D., Wu, Y. & Huang, X. Regional development and inequality within city regions: A study of the Yangtze River Delta, China. *Geogr. Rev.* **113**(3), 359–385 (2023).

[3] Mukherjee, M. & Takara, K. Urban green space as a countermeasure to increasing urban risk and the UGS-3CC resilience framework. *Int. J. Disaster Risk Reduct.* **28**, 854–861 (2018).

[4] Rigolon, A. A complex landscape of inequity in access to urban parks: A literature review. *Landsc. Urban Plan.* **153**, 160–169 (2016).

[5] Peters, K. Being together in urban parks: Connecting public space, leisure, and diversity. *Leis. Sci.* **32**(5), 418–433 (2010).

[6] Whyte, W. H. *City: Rediscovering the Center* (University of Pennsylvania Press, 2012).

[7] Zhang, J. & Tan, P. Y. Demand for parks and perceived accessibility as key determinants of urban park use behavior. *Urban For. Urban Green.* **44**, 126420 (2019).

[8] Biernacka, M., Łaszkiewicz, E. & Kronenberg, J. Park availability, accessibility, and attractiveness in relation to the least and most vulnerable inhabitants. *Urban For. Urban Green.* **73**, 127585 (2022).

[9] Chen, L., Lu, Y., Ye, Y., Xiao, Y. & Yang, L. Examining the association between the built environment and pedestrian volume using street view images. *Cities* **127**, 103734 (2022).

[10] Chen, J. et al. Rethinking urban park accessibility in the context of demographic change: A population structure perspective. *Urban For. Urban Green.* **96**, 128334 (2024).

[11] Zhou, S., Chen, F. & Xu, Z. _Evaluating the accessibility of urban parks and waterfronts through online map services: A case study of Shaoxing, China_. *Urban For. Urban Green.* **77**, 127731 (2022).

[12] Kenyon, G. E. et al. Intra-urban house prices in Madrid following the financial crisis: An exploration of spatial inequality. *npj Urban Sustain.* **4**(1), 26 (2024).

[13] Yang, H., Jin, C. & Li, T. A paradox of economic benefit and social equity of green space in megacity: Evidence from Tianjin in China. *Sustain. Cities Soc.* **109**, 105530 (2024).

[14] Zhang, K. & Chen, M. Multi-method analysis of urban green space accessibility: Influences of land use, greenery types, and individual characteristics factors. *Urban For. Urban Green.* **96**, 128366 (2024).

[15] Shelhamer, E., Long, J. & Darrell, T. Fully convolutional networks for semantic segmentation. *IEEE Trans. Pattern Anal. Mach. Intell.* **39**(4), 640–651 (2016). 27244717 10.1109/TPAMI.2016.2572683

[16] Ma, G. et al. Impact of land-use mixing on the vitality of urban parks: Evidence from big data analysis in Suzhou, Yangtze River Delta region, China. *J. Urban Plan. Dev.* **149**(4), 04023045 (2023).

[17] Fuller, R. A., Irvine, K. N., & Gaston, K. J. Interactions between people and nature in urban environments. Urban Ecology. 134–171 (2010).

[18] Fan, P. et al. Walkability in urban landscapes: A comparative study of four large cities in China. *Landscape Ecol.* **33**, 323–340 (2018).

[19] Basu, S. & Nagendra, H. Perceptions of park visitors on access to urban parks and benefits of green spaces. *Urban For. Urban Green.* **57**, 126959 (2021).

[20] Wang, Y. et al. Exploring recreational ecosystem services and influencing factors in megaparks using mobile phone data—Evidence from the Yangtze River Delta region. *Ecol. Indic.* **165**, 112195 (2024).

[21] Na, L., Huang, Y. & Chen, S. How different indicators of internal and external greenness impact street crime? A comparative study in high-density urban areas. *Habitat Int.* **171**, 103759 (2026).

[22] Yu, P. et al. Capturing open space fragmentation in high–density cities: Towards sustainable open space planning. *Appl. Geogr.* **154**, 102927 (2023).

[23] Boakye-Dankwa, E. et al. Walking behaviour and patterns of perceived access to neighbourhood destinations in older adults from a low-density (Brisbane, Australia) and an ultra-dense city (Hong Kong, China). *Cities* **84**, 23–33 (2019).

[24] Li, M., Xue, F. & Yeh, A. G. Bi-objective analytics of 3D visual-physical nature exposures in high-rise high-density cities for landscape and urban planning. *Landsc. Urban Plan.* **233**, 104714 (2023).

[25] Wang, L., Wong, C. & Duan, X. _Urban growth and spatial restructuring patterns: The case of Yangtze River Delta Region, China_. *Environ. Plan. B Plan. Des.* **43**(3), 515–539 (2016).

[26] Völker, S. & Kistemann, T. The impact of blue space on human health and well-being–Salutogenetic health effects of inland surface waters: A review. *Int. J. Hyg. Environ. Health* **214**(6), 449–460 (2011). 21665536 10.1016/j.ijheh.2011.05.001

[27] Ruiz-Padillo, A. et al. Weighted assessment of barriers to walking in small cities: A Brazilian case. *Transp. Res. D Transp. Environ.* **109**, 103392 (2022).

[28] Chen, Y., Zhu, M., Lu, J., Zhou, Q. & Ma, W. Evaluation of ecological city and analysis of obstacle factors under the background of high-quality development: Taking cities in the Yellow River Basin as examples. *Ecol. Indic.* **118**, 106771 (2020).

[29] Ma, X. et al. Measuring human perceptions of streetscapes to better inform urban renewal: A perspective of scene semantic parsing. *Cities* **110**, 103086 (2021).

[30] Low, S., Taplin, D. & Scheld, S. *Rethinking Urban Parks: Public Space and Cultural Diversity* (University of Texas Press, 2005).

[31] De Chiara, J., & Koppelman, L. Urban planning and design criteria. (No Title). (1975).

[32] Xiao, Y., Wang, Z., Li, Z. & Tang, Z. An assessment of urban park access in Shanghai-implications for the social equity in urban China. *Landsc. Urban Plan.* **157**, 383–393 (2017).

[33] Larkin, A. & Hystad, P. Evaluating street view exposure measures of visible green space for health research. *J. Expo. Sci. Environ. Epidemiol.* **29**(4), 447–456 (2019). 29352209 10.1038/s41370-018-0017-1

[34] Long, Y. & Liu, L. How green are the streets? An analysis for central areas of Chinese cities using Tencent Street View. *PLoS ONE* **12**(2), e0171110 (2017). 28196071 10.1371/journal.pone.0171110 PMC5308808

[35] Luo, S., Xie, J. & Furuya, K. Assessing the preference and restorative potential of urban park blue space. *Land* **10**(11), 1233 (2021).

[36] Hess, P. M. Measures of connectivity [Streets: Old paradigm, new investment]. Places. 11(2) (1997).

[37] Ewing, R. & Handy, S. Measuring the unmeasurable: Urban design qualities related to walkability. *J. Urban Des.* **14**(1), 65–84 (2009).

[38] Stangl, P. The US pedestrian plan: Linking practice and research. *Plan. Pract. Res.* **26**(3), 289–305 (2011).

[39] Dill, J. Measuring network connectivity for bicycling and walking. In *83rd Annual Meeting of the Transportation Research Board*, 11–15 (2004).

[40] Forman, R. T. T. *Land Mosaics: The Ecology of Landscapes and Regions* (Cambridge University Press, 2014).

[41] Wolch, J. R., Byrne, J. & Newell, J. P. Urban green space, public health, and environmental justice: The challenge of making cities ‘just green enough’. *Landsc. Urban Plan.* **125**, 234–244 (2014).

[42] Siegel, F. R. *Cities and mega-cities: problems and solution strategies* (Springer, 2019).

[43] Tonkiss, F. *Cities by Design: The Social Life of Urban Form* (Wiley, 2014).

[44] Baobeid, A., Koç, M. & Al-Ghamdi, S. G. Walkability and its relationships with health, sustainability, and livability: Elements of physical environment and evaluation frameworks. *Front. Built Environ.* **7**, 721218 (2021).

[45] Xu, J., Wang, J., Zuo, X. & Han, X. Spatial quality optimization analysis of streets in historical urban areas based on street view perception and multisource data. *J. Urban Plan. Dev.* **150**(4), 05024036 (2024).

[46] Li, W., Cai, Z. & Jin, L. Urban green land use efficiency of resource-based cities in China: Multidimensional measurements, spatial-temporal changes, and driving factors. *Sustain. Cities Soc.* **104**, 105299 (2024).

